# Uremic encephalopathy in critical care: does it exist and does renal replacement therapy improve outcome?

**DOI:** 10.1186/2197-425X-3-S1-A468

**Published:** 2015-10-01

**Authors:** M de Mos, EJ Hoorn, D Dippel, J van Bommel

**Affiliations:** Erasmus MC University Medical Center, Anesthesiology, Rotterdam, the Netherlands; Erasmus MC University Medical Center, Nephrology, Rotterdam, the Netherlands; Erasmus MC University Medical Center, Neurology, Rotterdam, the Netherlands; Erasmus MC University Medical Center, Intensive Care, Rotterdam, the Netherlands

## Introduction

Neurological disturbances and acute kidney injury (AKI) commonly co-exist in critically ill patients. However, it is unclear if AKI directly contributes to neurological disturbances (uremic encephalopathy) or if neurological disturbances and AKI are both reflections of multi-organ failure. Furthermore, it is unclear if renal replacement therapy (RRT) improves outcome, especially if other indications for RRT are absent.

## Objectives

To analyze the incidence, characteristics, effect of RRT, and outcomes in patients with neurological disturbances and AKI.

## Methods

During the study-period 2012-2013 at an university hospital intensive care unit (ICU) in The Netherlands, we retrospectively reviewed all neurology consults (n = 803) and then selected patients with concomitant AKI. Patients with intracranial pathology were excluded. After detailed analysis by a neurologist and nephrologist, patients were classified as neurological disturbances *with* AKI (NWH, neurological status attributable to several causes, including AKI) or neurological disturbances *due to* AKI (NDA, no other explanation for the neurological status than AKI).

## Results

The incidence of neurological disturbances and AKI was 6.6 per 1,000 ICU admissions

(95% CI 4.7 - 9.1). Of the 35 patients with neurological disturbances and AKI, NWA was four times more common than NDA (28 vs. 7 patients). Patients with NDA were more uremic (serum urea 42 vs. 23 mmol/l, p = 0.005), but had lower sequential organ failure (SOFA) scores (11.6 vs. 13.8, p = 0.04). RRT was started in 71% of NDA cases (in two patients coma was the only indication) and in 46% of NWA cases (p = 0.2). 28-day mortality was 57% in the NDA cases and 82% in the NWA cases (p = 0.2). Patients receiving RRT did not have better outcomes than patients not receiving RRT (neurological improvement 33 vs. 41%; 28-day mortality 83 vs. 69%; p > 0.05 for both).

## Conclusions

In critically ill patients with neurological disturbances and AKI, AKI is usually only one of the possible factors contributing to the neurological status. The combination of AKI and neurological disturbances signals a poor prognosis with RRT having little effect on neurological improvement or mortality.
